# *catena*-Poly[[(2-{1-[2-(2-amino­ethyl­amino)ethyl­imino]­eth­yl}-5-methoxy­phenolato-κ^4^ *N*,*N*′,*N*′′,*O*)copper(II)]-μ-nitrato-κ^2^ *O*:*O*′]

**DOI:** 10.1107/S1600536808021880

**Published:** 2008-07-23

**Authors:** Suwen Wang, Zhongfang Li, Xutao Wang, Xianjin Yu

**Affiliations:** a College of Chemical Engineering, Shandong University of Technology, Zibo 255049, People’s Republic of China

## Abstract

In the title compound, [Cu(C_13_H_20_N_3_O_2_)(NO_3_)]_*n*_, the Cu^II^ atom is chelated by the Schiff base ligand *via* three N atoms and one O atom lying in an approximate square plane (r.m.s. deviation = 0.04 Å). The complex mol­ecules are linked into a polymeric chain by bridging nitrate anions, forming axial Cu—O bonds of 2.535 (6) and 2.676 (7) Å, completing a distorted octa­hedral coordination geometry. The NH groups of the ligand form hydrogen bonds to the nitrate anions.

## Related literature

For related literature, see: Garnovskii *et al.* (1993 ▶); Huang *et al.* (2002 ▶); Bhadbhade & Srinivas (1993 ▶); Bunce *et al.* (1998 ▶).
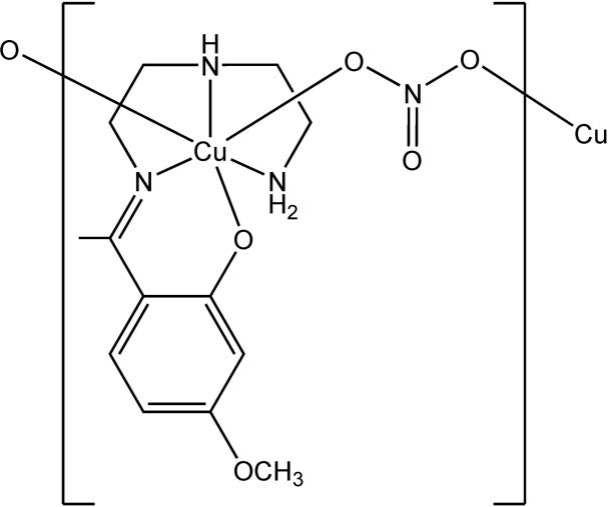


## Experimental

### 

#### Crystal data

[Cu(C_13_H_20_N_3_O_2_)(NO_3_)]
*M* *_r_* = 375.87
Triclinic, 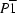

*a* = 7.2012 (10) Å
*b* = 10.095 (2) Å
*c* = 11.581 (2) Å
α = 69.15 (2)°
β = 89.73 (2)°
γ = 89.95 (2)°
*V* = 786.8 (3) Å^3^
*Z* = 2
Mo *K*α radiation
μ = 1.42 mm^−1^
*T* = 293 (2) K
0.43 × 0.28 × 0.22 mm

#### Data collection

Bruker APEXII CCD diffractometer
Absorption correction: multi-scan (*SADABS*; Bruker, 2001 ▶) *T*_min_ = 0.569, *T*_max_ = 0.730
4891 measured reflections
2739 independent reflections
1896 reflections with *I* > 2σ(*I*)
*R*_int_ = 0.029

#### Refinement

*R*[*F* ^2^ > 2σ(*F* ^2^)] = 0.060
*wR*(*F* ^2^) = 0.160
*S* = 1.00
2739 reflections
213 parameters
1 restraint
H atoms treated by a mixture of independent and constrained refinement
Δρ_max_ = 0.91 e Å^−3^
Δρ_min_ = −0.42 e Å^−3^

### 

Data collection: *APEX2* (Bruker, 2004 ▶); cell refinement: *SAINT-Plus* (Bruker, 2001 ▶); data reduction: *SAINT-Plus*; program(s) used to solve structure: *SHELXS97* (Sheldrick, 2008 ▶); program(s) used to refine structure: *SHELXL97* (Sheldrick, 2008 ▶); molecular graphics: *SHELXTL* (Sheldrick, 2008 ▶); software used to prepare material for publication: *SHELXTL*.

## Supplementary Material

Crystal structure: contains datablocks global, I. DOI: 10.1107/S1600536808021880/bi2290sup1.cif

Structure factors: contains datablocks I. DOI: 10.1107/S1600536808021880/bi2290Isup2.hkl

Additional supplementary materials:  crystallographic information; 3D view; checkCIF report

## Figures and Tables

**Table 1:** Hydrogen-bond geometry (Å, °)

*D*—H⋯*A*	*D*—H	H⋯*A*	*D*⋯*A*	*D*—H⋯*A*
N2—H1⋯O5^i^	0.90 (1)	2.15 (2)	3.013 (8)	161 (6)
N2—H1⋯O3^i^	0.90 (1)	2.65 (6)	3.134 (8)	115 (5)
N4—H4*A*⋯O2^ii^	0.90	2.43	3.316 (9)	168
N4—H4*B*⋯O3^ii^	0.90	2.29	3.157 (8)	162
N4—H4*B*⋯O4^ii^	0.90	2.66	3.175 (9)	118

Symmetry codes: (i) 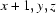; (ii) 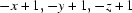.

**Fig. 1.**
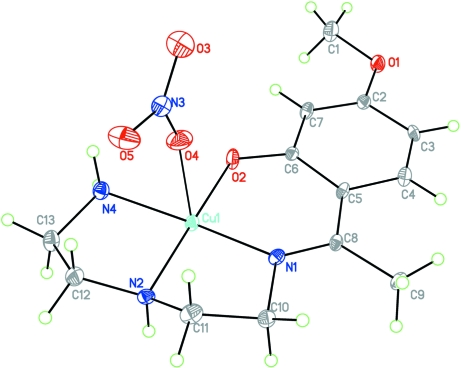
The asymmetric unit of the title compound drawn with 30% probability displacement ellipsoids for the non-hydrogen atoms.

## supplementary crystallographic information

### Comment

Schiff bases have been studied as ligands for a long time due to instant and enduring popularity from their easy synthesis and versatility in complexes. They play an important role in the development of coordination chemistry as well as inorganic biochemistry, catalysis, optical materials and so on (Garnovskii *et al.*, 1993; Huang *et al.*, 2002). Considerable attention has been focused on the syntheses and structures of Cu^II^ and Ni^II^ complexes. The Ni^II^ complexes with multidentate Schiff-base ligands have aroused particular interest because Ni can exhibit several oxidation states and may provide the basis of models for active sites of biological systems. On the other hand, the main attention in the optically active Schiff-base complexes is concentrated on their catalytic abilities in stereoselective synthesis (Bhadbhade & Srinivas, 1993; Bunce *et al.*, 1998).

### Experimental

A mixture of copper(II) nitrate hemi(pentahydrate) (1 mmol) and *N*-(2-hydroxy-4-methoxybenzyl)bisethylenetriamine (1 mmol) in 20 ml methanol was refluxed for two hours. The resulting solution was cooled and filtered and the filtrate was evaporated naturally at room temperature. Two day later, blue blocks were obtained with a yield of 16 %. Elemental analysis calculated: C 41.60, H 5.07, N 14.93 %; found: C 41.51, H 5.08, N 14.85 %.

### Refinement

H atoms bound to C atoms were placed in calculated positions with C—H = 0.93–0.97 Å and refined as riding with *U*_iso_(H) = 1.2 or 1.5*U*_eq_(C). The H atoms bound to N4 were also placed in calculated positions with N—H = 0.90 Å and allowed to ride with *U*_iso_(H) = 1.2*U*_eq_(N). Atom H1 was located in a difference Fourier map and its position was refined with the N—H distance restrained to 0.90 (1) Å and with *U*_iso_ = 0.05 Å^2^.

### Crystal data

**Table:**

[Cu(C_13_H_20_N_3_O_2_)(NO_3_)]	*Z* = 2
*M**_r_* = 375.87	*F*_000_ = 390
Triclinic, *P*1	*D*_x_ = 1.587 Mg m^−^^3^
Hall symbol: -P 1	Mo *K*α radiation λ = 0.71073 Å
*a* = 7.2012 (10) Å	Cell parameters from 2739 reflections
*b* = 10.095 (2) Å	θ = 2.2–25.0º
*c* = 11.581 (2) Å	µ = 1.42 mm^−^^1^
α = 69.15 (2)º	*T* = 293 (2) K
β = 89.73 (2)º	Block, blue
γ = 89.95 (2)º	0.43 × 0.28 × 0.22 mm
*V* = 786.8 (3) Å^3^	

### Data collection

**Table:**

Bruker APEXII CCD diffractometer	2739 independent reflections
Radiation source: fine-focus sealed tube	1896 reflections with *I* > 2σ(*I*)
Monochromator: graphite	*R*_int_ = 0.029
*T* = 293(2) K	θ_max_ = 25.0º
φ and ω scans	θ_min_ = 2.2º
Absorption correction: multi-scan(SADABS; Bruker, 2001)	*h* = −8→8
*T*_min_ = 0.569, *T*_max_ = 0.730	*k* = −11→12
4891 measured reflections	*l* = −13→13

### Refinement

**Table:**

Refinement on *F*^2^	Secondary atom site location: difference Fourier map
Least-squares matrix: full	Hydrogen site location: inferred from neighbouring sites
*R*[*F*^2^ > 2σ(*F*^2^)] = 0.060	H atoms treated by a mixture of independent and constrained refinement
*wR*(*F*^2^) = 0.160	*w* = 1/[σ^2^(*F*_o_^2^) + (0.107*P*)^2^ + 0.9393*P*] where *P* = (*F*_o_^2^ + 2*F*_c_^2^)/3
*S* = 1.00	(Δ/σ)_max_ = 0.016
2739 reflections	Δρ_max_ = 0.91 e Å^−^^3^
213 parameters	Δρ_min_ = −0.42 e Å^−^^3^
1 restraint	Extinction correction: none
Primary atom site location: structure-invariant direct methods	

### Special details

**Table:**

Geometry. All esds (except the esd in the dihedral angle between two l.s. planes) are estimated using the full covariance matrix. The cell esds are taken into account individually in the estimation of esds in distances, angles and torsion angles; correlations between esds in cell parameters are only used when they are defined by crystal symmetry. An approximate (isotropic) treatment of cell esds is used for estimating esds involving l.s. planes.
Refinement. Refinement of *F*^2^ against ALL reflections. The weighted *R*-factor *wR* and goodness of fit *S* are based on *F*^2^, conventional *R*-factors *R* are based on *F*, with *F* set to zero for negative *F*^2^. The threshold expression of *F*^2^ > σ(*F*^2^) is used only for calculating *R*-factors(gt) *etc*. and is not relevant to the choice of reflections for refinement. *R*-factors based on *F*^2^ are statistically about twice as large as those based on *F*, and *R*- factors based on ALL data will be even larger.

### Fractional atomic coordinates and isotropic or equivalent isotropic displacement parameters (Å^2^)

**Table:**

	*x*	*y*	*z*	*U*_iso_*/*U*_eq_	
Cu1	0.72893 (13)	0.60509 (7)	0.61951 (6)	0.0396 (3)	
C2	0.7556 (9)	0.9968 (6)	0.1737 (5)	0.0428 (13)	
C3	0.7606 (11)	1.1062 (6)	0.2212 (6)	0.0533 (16)	
H3	0.7675	1.2001	0.1682	0.064*	
C4	0.7551 (10)	1.0737 (7)	0.3470 (6)	0.0511 (16)	
H4	0.7594	1.1475	0.3772	0.061*	
C5	0.7434 (8)	0.9336 (6)	0.4323 (5)	0.0355 (12)	
C6	0.7281 (8)	0.8240 (6)	0.3825 (5)	0.0379 (12)	
C7	0.7387 (9)	0.8620 (6)	0.2511 (6)	0.0430 (14)	
H7	0.7337	0.7905	0.2181	0.052*	
C8	0.7388 (8)	0.9117 (6)	0.5646 (5)	0.0364 (12)	
C9	0.7516 (10)	1.0377 (7)	0.6051 (6)	0.0503 (15)	
H9A	0.6518	1.0342	0.6614	0.075*	
H9B	0.7428	1.1235	0.5342	0.075*	
H9C	0.8682	1.0357	0.6455	0.075*	
C10	0.7303 (11)	0.7615 (7)	0.7821 (5)	0.0519 (16)	
H10A	0.6546	0.8307	0.8011	0.062*	
H10B	0.8577	0.7716	0.8046	0.062*	
C11	0.6598 (11)	0.6117 (8)	0.8535 (6)	0.0625 (19)	
H11A	0.6895	0.5850	0.9404	0.075*	
H11B	0.5260	0.6079	0.8456	0.075*	
C12	0.6772 (13)	0.3710 (8)	0.8405 (7)	0.0637 (19)	
H12A	0.5426	0.3719	0.8441	0.076*	
H12B	0.7233	0.3152	0.9219	0.076*	
C13	0.7406 (11)	0.3068 (7)	0.7474 (6)	0.0562 (17)	
H13A	0.8729	0.2881	0.7557	0.067*	
H13B	0.6766	0.2177	0.7620	0.067*	
N1	0.7187 (7)	0.7844 (5)	0.6477 (4)	0.0402 (11)	
N2	0.7485 (8)	0.5147 (5)	0.8029 (4)	0.0415 (11)	
H1	0.871 (2)	0.517 (7)	0.816 (6)	0.050*	
N3	0.2005 (9)	0.5570 (6)	0.6866 (6)	0.0550 (14)	
N4	0.6991 (8)	0.4070 (5)	0.6214 (5)	0.0500 (13)	
H4A	0.5822	0.3937	0.6006	0.060*	
H4B	0.7773	0.3921	0.5664	0.060*	
O1	0.7593 (8)	1.0387 (5)	0.0493 (4)	0.0616 (13)	
O2	0.7129 (9)	0.6914 (4)	0.4468 (4)	0.0633 (15)	
O3	0.0805 (9)	0.5939 (7)	0.6146 (6)	0.0869 (18)	
O4	0.3604 (9)	0.5871 (7)	0.6519 (7)	0.096 (2)	
O5	0.1656 (9)	0.4935 (8)	0.7984 (6)	0.098 (2)	
C1	0.7476 (17)	0.9303 (8)	−0.0054 (6)	0.080 (3)	
H1A	0.8518	0.8674	0.0206	0.121*	
H1B	0.7488	0.9741	−0.0938	0.121*	
H1C	0.6346	0.8776	0.0207	0.121*	

### Atomic displacement parameters (Å^2^)

**Table:**

	*U*^11^	*U*^22^	*U*^33^	*U*^12^	*U*^13^	*U*^23^
Cu1	0.0575 (5)	0.0319 (4)	0.0322 (4)	−0.0024 (3)	0.0031 (3)	−0.0149 (3)
C2	0.040 (3)	0.038 (3)	0.048 (3)	−0.004 (2)	0.000 (3)	−0.013 (3)
C3	0.076 (5)	0.029 (3)	0.046 (3)	0.007 (3)	−0.016 (3)	0.000 (2)
C4	0.070 (4)	0.035 (3)	0.048 (3)	−0.004 (3)	0.013 (3)	−0.015 (3)
C5	0.032 (3)	0.033 (3)	0.048 (3)	−0.006 (2)	0.013 (2)	−0.022 (2)
C6	0.045 (3)	0.033 (3)	0.035 (3)	−0.006 (2)	−0.003 (2)	−0.011 (2)
C7	0.050 (4)	0.038 (3)	0.048 (3)	−0.005 (3)	0.015 (3)	−0.025 (3)
C8	0.031 (3)	0.030 (3)	0.051 (3)	0.001 (2)	0.006 (2)	−0.018 (2)
C9	0.058 (4)	0.046 (3)	0.060 (4)	−0.001 (3)	−0.001 (3)	−0.034 (3)
C10	0.077 (5)	0.048 (3)	0.037 (3)	0.001 (3)	0.002 (3)	−0.022 (3)
C11	0.064 (5)	0.078 (5)	0.053 (4)	−0.009 (4)	0.010 (3)	−0.033 (4)
C12	0.087 (6)	0.053 (4)	0.053 (4)	−0.005 (4)	−0.007 (4)	−0.021 (3)
C13	0.072 (5)	0.044 (4)	0.053 (4)	0.000 (3)	0.016 (3)	−0.020 (3)
N1	0.046 (3)	0.044 (3)	0.038 (2)	−0.001 (2)	0.005 (2)	−0.025 (2)
N2	0.046 (3)	0.049 (3)	0.032 (2)	−0.003 (2)	0.002 (2)	−0.018 (2)
N3	0.055 (4)	0.055 (3)	0.057 (4)	−0.002 (3)	0.004 (3)	−0.022 (3)
N4	0.055 (3)	0.046 (3)	0.049 (3)	−0.005 (2)	0.006 (2)	−0.017 (2)
O1	0.098 (4)	0.042 (2)	0.040 (2)	−0.006 (2)	−0.007 (2)	−0.0085 (19)
O2	0.125 (5)	0.029 (2)	0.036 (2)	−0.005 (2)	0.000 (2)	−0.0107 (17)
O3	0.065 (4)	0.108 (5)	0.078 (4)	−0.005 (3)	0.020 (3)	−0.022 (3)
O4	0.060 (4)	0.100 (5)	0.147 (6)	0.018 (3)	−0.040 (4)	−0.067 (5)
O5	0.071 (4)	0.137 (6)	0.076 (4)	0.003 (4)	−0.010 (3)	−0.026 (4)
C1	0.155 (9)	0.052 (4)	0.038 (3)	−0.008 (5)	−0.003 (4)	−0.020 (3)

### Geometric parameters (Å, °)

**Table:**

Cu1—N1	1.952 (5)	C10—C11	1.529 (10)
Cu1—N2	1.997 (5)	C10—H10A	0.970
Cu1—N4	2.004 (5)	C10—H10B	0.970
Cu1—O2	1.880 (4)	C11—N2	1.453 (9)
Cu1—O3^i^	2.535 (6)	C11—H11A	0.970
Cu1—O4	2.676 (7)	C11—H11B	0.970
C2—C7	1.342 (8)	C12—N2	1.451 (9)
C2—O1	1.350 (7)	C12—C13	1.511 (10)
C2—C3	1.398 (9)	C12—H12A	0.970
C3—C4	1.374 (9)	C12—H12B	0.970
C3—H3	0.930	C13—N4	1.481 (9)
C4—C5	1.410 (8)	C13—H13A	0.970
C4—H4	0.930	C13—H13B	0.970
C5—C6	1.422 (8)	N2—H1	0.90 (1)
C5—C8	1.468 (8)	N3—O3	1.169 (9)
C6—O2	1.284 (7)	N3—O4	1.220 (9)
C6—C7	1.433 (8)	N3—O5	1.248 (8)
C7—H7	0.930	N4—H4A	0.900
C8—N1	1.310 (7)	N4—H4B	0.900
C8—C9	1.508 (8)	O1—C1	1.449 (9)
C9—H9A	0.960	C1—H1A	0.960
C9—H9B	0.960	C1—H1B	0.960
C9—H9C	0.960	C1—H1C	0.960
C10—N1	1.493 (7)		
			
O2—Cu1—N1	93.98 (19)	N2—C11—H11A	110.0
O2—Cu1—N2	179.4 (3)	C10—C11—H11A	110.0
N1—Cu1—N2	85.7 (2)	N2—C11—H11B	110.0
O2—Cu1—N4	95.0 (2)	C10—C11—H11B	110.0
N1—Cu1—N4	167.3 (2)	H11A—C11—H11B	108.4
N2—Cu1—N4	85.4 (2)	N2—C12—C13	108.5 (6)
O2—Cu1—O4	93.8 (3)	N2—C12—H12A	110.0
N1—Cu1—O4	87.8 (2)	C13—C12—H12A	110.0
N2—Cu1—O4	86.7 (2)	N2—C12—H12B	110.0
N4—Cu1—O4	82.8 (2)	C13—C12—H12B	110.0
C7—C2—O1	124.9 (5)	H12A—C12—H12B	108.4
C7—C2—C3	119.6 (6)	N4—C13—C12	109.0 (6)
O1—C2—C3	115.4 (5)	N4—C13—H13A	109.9
C4—C3—C2	119.5 (6)	C12—C13—H13A	109.9
C4—C3—H3	120.3	N4—C13—H13B	109.9
C2—C3—H3	120.3	C12—C13—H13B	109.9
C3—C4—C5	123.1 (6)	H13A—C13—H13B	108.3
C3—C4—H4	118.5	C8—N1—C10	120.5 (5)
C5—C4—H4	118.5	C8—N1—Cu1	126.7 (4)
C4—C5—C6	116.8 (5)	C10—N1—Cu1	111.3 (4)
C4—C5—C8	118.2 (5)	C12—N2—C11	118.0 (6)
C6—C5—C8	124.9 (5)	C12—N2—Cu1	108.8 (4)
O2—C6—C5	124.9 (5)	C11—N2—Cu1	106.1 (4)
O2—C6—C7	116.7 (5)	C12—N2—H1	112 (4)
C5—C6—C7	118.3 (5)	C11—N2—H1	108 (4)
C2—C7—C6	122.5 (5)	Cu1—N2—H1	103 (4)
C2—C7—H7	118.7	O3—N3—O4	119.1 (6)
C6—C7—H7	118.7	O3—N3—O5	120.6 (7)
N1—C8—C5	120.8 (5)	O4—N3—O5	120.2 (7)
N1—C8—C9	119.6 (5)	C13—N4—Cu1	108.5 (4)
C5—C8—C9	119.6 (5)	C13—N4—H4A	110.0
C8—C9—H9A	109.5	Cu1—N4—H4A	110.0
C8—C9—H9B	109.5	C13—N4—H4B	110.0
H9A—C9—H9B	109.5	Cu1—N4—H4B	110.0
C8—C9—H9C	109.5	H4A—N4—H4B	108.4
H9A—C9—H9C	109.5	C2—O1—C1	117.9 (5)
H9B—C9—H9C	109.5	C6—O2—Cu1	127.1 (4)
N1—C10—C11	107.4 (5)	N3—O4—Cu1	167.6 (6)
N1—C10—H10A	110.2	O1—C1—H1A	109.5
C11—C10—H10A	110.2	O1—C1—H1B	109.5
N1—C10—H10B	110.2	H1A—C1—H1B	109.5
C11—C10—H10B	110.2	O1—C1—H1C	109.5
H10A—C10—H10B	108.5	H1A—C1—H1C	109.5
N2—C11—C10	108.5 (6)	H1B—C1—H1C	109.5

Symmetry codes: (i) *x*+1, *y*, *z*.

### Hydrogen-bond geometry (Å, °)

**Table:**

*D*—H···*A*	*D*—H	H···*A*	*D*···*A*	*D*—H···*A*
N2—H1···O5^i^	0.90 (1)	2.15 (2)	3.013 (8)	161 (6)
N2—H1···O3^i^	0.90 (1)	2.65 (6)	3.134 (8)	115 (5)
N4—H4A···O2^ii^	0.90	2.43	3.316 (9)	168
N4—H4B···O3^ii^	0.90	2.29	3.157 (8)	162
N4—H4B···O4^ii^	0.90	2.66	3.175 (9)	118

Symmetry codes: (i) *x*+1, *y*, *z*; (ii) −*x*+1, −*y*+1, −*z*+1.
